# Association between parent-reported salt-related behaviors and estimated urinary salt excretion: a cross-sectional study of health checkups in 4-year-old children

**DOI:** 10.1265/ehpm.25-00076

**Published:** 2025-05-24

**Authors:** Takafumi Abe, Minoru Isomura, Shozo Yano

**Affiliations:** 1 Center for Community-Based Healthcare Research and Education (CoHRE), Head Office for Research and Academic Information, Shimane University, Izumo, Shimane, Japan; 2 Faculty of Human Sciences, Shimane University, Matsue, Shimane, Japan; 3 Department of Laboratory Medicine, Faculty of Medicine, Shimane University, Izumo, Shimane, Japan

**Keywords:** Health policy, Preschool, Salt intake

## Abstract

Although salt-related behaviors may influence urinary salt excretion in early childhood, this relationship remains unclear. This study aimed to examine salt-related behaviors using data from a salt check sheet and urinary salt excretion parameters using spot urine samples from 4-year-old children. This cross-sectional study included all 4-year-old children who underwent health checkups in Ohnan Town, Shimane Prefecture. The study sample consisted of 109 children (49 boys). Measures from spot urine samples included estimated salt excretion (g/day) and the sodium-potassium (Na/K) ratio. Salt-related behaviors were assessed using a salt check sheet that was completed by the parents or guardians. The associations between salt-related behaviors and urinary salt excretion parameters were analyzed using a generalized linear model. The median (M) and interquartile range (IQR) for urinary measures in 4-year-old children were as follows: estimated salt excretion (M = 4.4, IQR: 3.3–6.2) and Na/K ratio (M = 2.3, IQR: 1.4–3.3). The low frequency of consumption of high-salt foods (“such as pickles, pickled plums, etc.” and “noodles such as *udon* and *ramen*”) was associated with low salt excretion and low Na/K ratio. However, in the case of “consumption of *udon*, *ramen*, or other soups”, the Na/K ratio was higher for “About half a bowl” and “Some” than for “An entire bowl.” Additionally, for “eating out or having convenience-store-bought bento (lunch plate) for lunch”, the Na/K ratio was higher for “No” than for “Almost every day.” In conclusion, the frequency of high-sodium food intake was associated with both urinary sodium excretion and the Na/K ratio in 4-year-old children. Longitudinal investigations using the 24-hour urine collection method are needed to confirm these salt-related behaviors.

**Supplementary information:**

The online version contains supplementary material available at https://doi.org/10.1265/ehpm.25-00076.

**Dear Editor:**

The World Health Organization states that the recommended maximum sodium intake of 2 g per day for adults should be adjusted for children, considering the energy requirements of children compared to adults [1]. The Japanese Ministry of Health, Labour and Welfare recommends that the daily salt intake for children aged 3–5 years be <3 g [2]. The Japanese Society of Hypertension recently released a consensus statement regarding the practical application and target values of the urine sodium-to-potassium (Na/K) ratio for Japanese populations [3]. The Na/K ratio, derived from the sodium and potassium levels in spot urine samples, is considered less invasive and imposes minimal burden on participants. As a result, the Na/K ratio is expected to be adopted as a new health-related parameter. Providing appropriate salt reduction education from a young age to establish healthy diet is crucial for preventing hypertension throughout life. Therefore, the relationship between daily eating behaviors and salt intake needs to be clarified.

Previous studies have investigated the relationship between urinary salt excretion parameters based on spot urine samples from Japanese infants using a dietary questionnaire [4–6]. These studies found that processed meat products, vegetables, fruits, and seasoning and spices were associated with salt-related parameters. However, dietary habits vary by region, making it important to understand these differences. Investigating salt-related behaviors is not easy; the salt check sheet developed by Tsuchihashi et al. has been used in clinical education and research [7]. Studies targeting children have surveyed Japanese elementary or junior high school students and reported correlations with self-reported salt intake [8]. However, research on the relationship between salt-related behaviors using a salt check sheet and urinary salt excretion parameters in early childhood is lacking. Therefore, this study aimed to elucidate salt-related behaviors and urinary salt excretion parameters by analyzing urine samples collected during annual health checkups of 4-year-old children.

This study included children who participated in health checkups for 4-year-olds conducted in Ohnan Town (population 10,163, area 419.29 km^2^ in 2020), Shimane Prefecture, Japan, between May 2022 and March 2024. The municipality invited all children residing in Ohnan Town to participate in the health checkup in the year they turned 4. Additional tests were conducted alongside the routine health checkups.

Before including the participants in the study, written and verbal explanations of the research were provided to parents or guardians, and informed assent and consent were obtained. The study protocol was approved by the Research Ethics Committee for Human Subjects of Shimane University Faculty of Human Sciences (#2019-21). All procedures were performed in accordance with the tenets of the Declaration of Helsinki.

The inclusion criteria for the study participants were as follows: (1) those undergoing health check-ups, (2) those whose parents granted consent for participation, and (3) those who completed a urine test. The exclusion criteria were as follows: (1) those whose parents refused to consent, and (2) those with kidney disease as reported by their guardians. During the survey period, 122 children were eligible for participation, and the parents of 110 (90.2%) provided consent. One child was excluded owing to the inability to collect a urine sample, resulting in a final analysis sample of 109 children (89.3%).

Spot urine samples were collected during the afternoon health checkups. The samples were sent to Healthcare Systems Co., Ltd. (Aichi, Japan) for analysis. Urinary sodium (Na, mmol/L), potassium (K, mmol/L), and creatinine (mmol/dL) levels were measured. Estimated salt excretion was calculated using the following formula proposed in a previous study [4]:

$$\begin{array}{rl} & \text{Estimated salt excretion}\\ & \;=0.0585*300*\text{Na concentration}\\ & \;/(10*\text{creatinine concentration}) \end{array}$$

As sodium was measured in mEq/L and creatinine in mg/dL, creatinine values were multiplied by 10. The Na/K ratio was calculated by dividing urinary sodium concentration by urinary potassium concentration.

Salt-related behaviors were assessed using the salt check sheet [7, 9] that was completed by the parents or guardians. Sex and age in months (calculated based on the date of birth) were obtained from health checkup records. Height and weight were obtained from health checkup records. Body mass index (BMI) was calculated by dividing weight (kg) by the square of height (m^2^). BMI was categorized as thinness, healthy weight, or overweight/obesity, based on age-specific cut-off values established by the International Obesity Task Force [10].

The distribution of urinary salt excretion parameters was presented by a salt check sheet. Generalized linear models were used to examine whether each item of a salt check sheet as salt related behaviors was associated with urinary salt excretion parameters adjusted for sex, age months, BMI, and year of survey. The analysis results were presented after transforming the dependent variable using the natural logarithm. Statistical significance was set at 5%.

The characteristics of the participants and the response frequencies to the salt check sheet are shown in Supplemental Tables 1 and 2, respectively. Table 1 shows percentiles of urinary salt excretion parameters by each item of a salt check sheet. Table 2 indicates the association between each salt related behavior and urinary salt excretion parameters. Lower estimated salt excretion or Na/K ratios were observed for infrequent intake of “pickles, pickled plums, etc.” than for the intake frequency of more than twice a day. Specifically, those who consumed them once a day, two-to-three times a week, or hardly at all showed significantly lower values. Similarly, lower salt excretion or Na/K ratios were found among participants who ate noodles (e.g., *udon*, *ramen*) two or three times a week or less than once a week compared to those who consumed them almost every day. In contrast, a higher Na/K ratio was observed among those who consumed approximately half a bowl or a small portion of soup-based dishes (e.g., *udon* or *ramen* soups) than among those who consumed an entire bowl. Regarding lunch habits, individuals who did not eat out or purchase convenience-store lunches had a significantly higher Na/K ratio than those who did so almost every day.

**Table 1:** Salt-related parameters for each item on the salt check sheet

**Salt check-sheet items**	**Response options, median (IQR)**			
**Frequency of high-salt diet intake**				
*Miso* soup, other soup, etc.	More than 2 bowls a day	About 1 bowl a day	2–3 bowls a week	Hardly eat
Estimated salt excretion, g/day	3.6 (3.0, 7.6)	4.4 (3.5, 6.0)	4.8 (3.5, 5.8)	3.7 (3.2, 6.2)
Sodium-to-potassium ratio	2.2 (1.4, 3.0)	2.2 (1.1, 3.4)	2.4 (1.7, 2.9)	2.6 (1.4, 3.9)
Pickles, pickled plums, etc.	More than twice a day	About once a day	2–3 times a week	Hardly eat
Estimated salt excretion, g/day	7.7 (5.9, 9.5)	3.4 (2.7, 6.4)	4.9 (3.7, 6.2)	4.2 (3.2, 5.8)
Sodium-to-potassium ratio	4.2 (3.7, 4.7)	2.5 (2.3, 3.4)	2.5 (2.0, 3.5)	2.0 (1.3, 2.8)
Fish-paste products such as *chikuwa* and *kamaboko*	Eat frequently	2–3 times a week	Hardly eat	
Estimated salt excretion, g/day	3.6 (1.9, 6.6)	4.2 (3.1, 5.3)	5.1 (3.5, 6.6)	
Sodium-to-potassium ratio	2.8 (1.3, 4.0)	1.9 (1.1, 2.9)	2.4 (1.5, 3.4)	
Opened and dried horse mackerel, *mirin*-seasoned dried fish, salted salmon, etc.	Eat frequently	2–3 times a week	Hardly eat	
Estimated salt excretion, g/day	1.2 (0.8, 5.3)	4.8 (3.4, 5.8)	4.4 (3.3, 6.2)	
Sodium-to-potassium ratio	1.1 (0.6, 2.9)	2.8 (1.4, 3.7)	2.1 (1.4, 2.6)	
Ham or sausage	Eat frequently	2–3 times a week	Hardly eat	
Estimated salt excretion, g/day	3.9 (3.5, 6.1)	4.7 (3.3, 6.2)	3.8 (2.9, 5.8)	
Sodium-to-potassium ratio	2.2 (1.2, 3.0)	2.5 (1.6, 3.4)	2.0 (1.2, 2.5)	
Noodles such as *udon* and *ramen*	Almost every day	2 or 3 bowls a week	Less than once a week	Don’t eat
Estimated salt excretion, g/day	N/A	4.9 (3.5, 6.3)	4.4 (3.2, 5.8)	8.1 (5.8, 10.5)
Sodium-to-potassium ratio	N/A	2.1 (1.2, 2.8)	2.4 (1.4, 3.4)	2.6 (2.1, 3.0)
*Senbei*, *okaki*, potato chips, etc.	Eat frequently	2–3 times a week	Hardly eat	
Estimated salt excretion, g/day	3.7 (2.4, 4.2)	4.8 (3.3, 6.2)	4.7 (3.4, 5.8)	
Sodium-to-potassium ratio	1.8 (1.4, 2.9)	2.3 (1.5, 3.5)	2.3 (1.4, 3.0)	
**Additional seasoning, frequency of eating out, and home-meal replacement**				
Frequency of seasoning with soy sauce, other sauces, etc.	Season frequently (almost each meal)	Once a day	Season sometimes	Don’t season
Estimated salt excretion, g/day	3.7 (3.6, 6.6)	4.9 (3.5, 6.3)	4.3 (3.3, 5.9)	4.7 (2.5, 6.0)
Sodium-to-potassium ratio	3.5 (2.4, 4.1)	2.5 (1.5, 3.5)	2.2 (1.5, 2.9)	1.8 (1.1, 3.0)
Consumption of *udon*, *ramen*, or other soups	An entire bowl	About half a bowl	Some	Litte
Estimated salt excretion, g/day	4.5 (2.7, 5.5)	4.6 (3.6, 5.8)	4.2 (3.2, 6.8)	4.5 (3.2, 6.2)
Sodium-to-potassium ratio	1.5 (1.1, 1.8)	2.1 (1.5, 3.4)	2.5 (1.9, 3.4)	2.2 (1.1, 2.8)
Eating out or having convenience-store-bought *bento* (lunch plate) for lunch	Almost every day	About 3 times a week	About once a week	No
Estimated salt excretion, g/day	3.5 (2.0, 3.7)	4.4 (3.7, 5.6)	4.1 (3.3, 5.8)	5.1 (3.3, 6.4)
Sodium-to-potassium ratio	1.2 (0.7, 1.8)	1.9 (1.5, 2.5)	2.2 (1.3, 3.4)	2.4 (1.6, 3.5)
Eating out or having ready-made side dishes for dinner	Almost every day	About 3 times a week	About once a week	No
Estimated salt excretion, g/day	1.9 (0.4, 3.4)	5.0 (3.3, 5.6)	4.0 (3.3, 5.8)	5.1 (3.7, 6.7)
Sodium-to-potassium ratio	1.3 (0.2, 2.3)	2.1 (1.3, 3.7)	2.3 (1.3, 2.8)	2.4 (1.5, 3.5)
**Taste of homemade dishes, amount of food**				
Taste of homemade dishes: comparison with those eaten out	Heavily salted	The same	Lightly salted	
Estimated salt excretion, g/day	6.3 (4.5, 6.7)	4.4 (3.3, 6.1)	4.4 (3.5, 5.9)	
Sodium-to-potassium ratio	5.1 (3.1, 5.1)	2.2 (1.4, 2.9)	2.4 (1.5, 3.4)	
Amount of food	More than others	The same as others	Less than others	
Estimated salt excretion, g/day	5.9 (3.7, 7.0)	4.2 (3.2, 5.8)	7.4 (4.9, 8.0)	
Sodium-to-potassium ratio	2.5 (1.7, 3.0)	2.2 (1.4, 3.4)	2.1 (1.0, 2.5)	

Interquartile range: IQR

**Table 2:** Association between salt-related parameters and each item on the salt check sheet

**Salt check-sheet items**	**Estimated salt excretion**				**Sodium-to-potassium ratio**			
**Frequency of high-salt diet intake**								
*Miso* soup, other soup, etc.	More than 2 bowls a day	About 1 bowl a day	2–3 bowls a week	Hardly eat	More than 2 bowls a day	About 1 bowl a day	2–3 bowls a week	Hardly eat
*β* (95% CI)	Ref	−0.05 (−0.35, 0.26)	−0.02 (−0.32, 0.28)	−0.03 (−0.48, 0.41)	Ref	−0.16 (−0.49, 0.15)	−0.05 (−0.34, 0.25)	0.18 (−0.36, 0.73)
Pickles, pickled plums, etc.	More than twice a day	About once a day	2–3 times a week	Hardly eat	More than twice a day	About once a day	2–3 times a week	Hardly eat
*β* (95% CI)	Ref	**−0.79** **(−1.33, −0.25)**	**−0.59** **(−0.99, −0.19)**	**−0.71** **(−1.12, −0.29)**	Ref	**−0.60** **(−1.12, −0.09)**	**−0.62** **(−0.92, −0.32)**	**−0.80** **(−1.12, −0.48)**
Fish-paste products such as *chikuwa* and *kamaboko*	Eat frequently	2–3 times a week	Hardly eat		Eat frequently	2–3 times a week	Hardly eat	
*β* (95% CI)	Ref	0.39 (−0.74, 1.52)	0.54 (−0.57, 1.66)		Ref	0.19 (−1.06, 1.43)	0.38 (−0.86, 1.61)	
Opened and dried horse mackerel, *mirin*-seasoned dried fish, salted salmon, etc.	Eat frequently	2–3 times a week	Hardly eat		Eat frequently	2–3 times a week	Hardly eat	
*β* (95% CI)	Ref	0.90 (−0.70, 2.48)	0.90 (−0.68, 2.48)		Ref	0.84 (−0.78, 2.47)	0.69 (−0.91, 2.29)	
Ham or sausage	Eat frequently	2–3 times a week	Hardly eat		Eat frequently	2–3 times a week	Hardly eat	
*β* (95% CI)	Ref	0.11 (−0.23, 0.46)	−0.03 (−0.42, 0.37)		Ref	0.29 (−0.09, 0.67)	0.002 (−0.42, 0.43)	
Noodles such as *udon* and *ramen*	Almost every day	2 or 3 bowls a week	Less than once a week	Don’t eat	Almost every day	2 or 3 bowls a week	Less than once a week	Don’t eat
*β* (95% CI)	Ref	**−0.50** **(−0.72, −0.28)**	**−0.57** **(−0.73, −0.41)**	0.10 (−0.31, 0.51)	Ref	**−0.66** **(−0.92, −0.40)**	**−0.52** **(−0.70, −0.34)**	−0.40 (−0.80, 0.004)
*Senbei*, *okaki*, potato chips, etc.	Eat frequently	2–3 times a week	Hardly eat		Eat frequently	2–3 times a week	Hardly eat	
*β* (95% CI)	Ref	0.35 (−0.03, 0.72)	0.36 (−0.02, 0.73)		Ref	0.17 (−0.27, 0.60)	0.23 (−0.19, 0.64)	
**Additional seasoning, frequency of eating out, and home-meal replacement**								
Frequency of seasoning with soy sauce, other sauces, etc.	Season frequently (almost each meal)	Once a day	Season sometimes	Don’t season	Season frequently (almost each meal)	Once a day	Season sometimes	Don’t season
*β* (95% CI)	Ref	−0.10 (−0.72, 0.52)	−0.15 (−0.75, 0.46)	−0.34 (−1.01, 0.34)	Ref	−0.12 (−0.87, 0.63)	−0.25 (−0.98, 0.48)	−0.35 (−1.17, 0.48)
Consumption of *udon*, *ramen*, or other soups	An entire bowl	About half a bowl	Some	Litte	An entire bowl	About half a bowl	Some	Litte
*β* (95% CI)	Ref	0.22 (−0.27, 0.72)	0.27 (−0.20, 0.74)	0.15 (−0.31, 0.62)	Ref	**0.43** (**0.06, 0.81)**	**0.59** (**0.29, 0.88)**	0.32 (−0.02, 0.65)
Eating out or having convenience-store-bought *bento* (lunch plate) for lunch	Almost every day	About 3 times a week	About once a week	No	Almost every day	About 3 times a week	About once a week	No
*β* (95% CI)	Ref	0.80 (−0.10, 1.71)	0.73 (−0.17, 1.63)	0.81 (−0.10, 1.71)	Ref	0.71 (−0.23, 1.64)	0.88 (−0.07, 1.83)	**0.98** **(0.05, 1.92)**
Eating out or having ready-made side dishes for dinner	Almost every day	About 3 times a week	About once a week	No	Almost every day	About 3 times a week	About once a week	No
*β* (95% CI)	Ref	1.25 (−0.24, 2.74)	1.22 (−0.26, 2.69)	1.35 (−0.12, 2.83)	Ref	1.12 (−0.64, 2.89)	1.15 (−0.58, 2.89)	1.29 (−0.45, 3.03)
**Taste of homemade dishes, amount of food**								
Taste of homemade dishes: comparison with those eaten out	Heavily salted	The same	Lightly salted		Heavily salted	The same	Lightly salted	
*β* (95% CI)	Ref	−0.21 (−0.73, 0.31)	−0.13 (−0.65, 0.39)		Ref	−0.49 (−1.45, 0.46)	−0.36 (−1.31, 0.58)	
Amount of food	More than others	The same as others	Less than others		More than others	The same as others	Less than others	
*β* (95% CI)	Ref	−0.23 (−0.51, 0.06)	0.05 (−0.41, 0.52)		Ref	−0.13 (−0.37, 0.12)	−0.41 (−0.87, 0.06)	

Unstandardized coefficient: B, confidence intervals: CI

The generalized linear model was applied to each item on the salt check sheet. Sex, age in months and body mass index, and year of survey were adjusted. The analysis results after transforming the dependent variable using the natural logarithm are presented. Bold values indicate significance levels below 5%.

Low-frequency intake of high-salt foods (e.g., pickles, pickled plums, and noodles such as *udon* or *ramen*) was associated with lower salt excretion and Na/K ratio. However, for soup consumption, the Na/K ratio was higher among those who consumed “About half a bowl” or “Some” than among those who consumed “An entire bowl.” Similarly, the Na/K ratio was higher among those who did not eat out or buy convenience-store bento than among those who did so almost daily.

Pickles, pickled plums, etc., and noodles such as *udon* and *ramen* are known as high-salt foods in Japan. The infrequent provision of such foods at home may contribute to reduced salt intake in young children. On the other hand, the low consumption of soups such as *udon* and absence of convenience store *bento* were associated with a high Na/K ratio. Previous studies found that processed meat products, vegetables, fruits, and seasoning and spices were associated with urinary salt excretion parameters [4–6]. However, our study focused on different factors compared to previous studies.

Although it is challenging to explain the relationship that contradicts the hypothesis, the possibility that parents’ perceptions of the amount of soup being incorrect cannot be ruled out. Particularly, the frequency of noodle consumption such as *udon* was associated, suggesting that there may be a lack of potassium-containing foods (i.e. vegetables and fruits) at those times. Morinaga et al. reported that vegetable or fruit consumption, which is a source of potassium, was associated with the Na/K ratio [4–6]. The questionnaire used in this study to assess salt-related behaviors has been employed in research involving elementary and junior high school students [8], and it was not originally developed to evaluate dietary behaviors in young children. This may have influenced the study results. Further investigations are needed to elucidate these findings.

This study had several limitations. First, the sample size was relatively small and limited to a single town, and this may have introduced sampling error and bias, thereby limiting the generalizability of the findings to other populations or regions. However, with a participation rate of 90.2%, the study achieved a high level of engagement from the target population, using a comprehensive survey. Secondly, the study was conducted in a single town, which may not have captured the diversity of dietary habits, environmental factors, and health conditions found in other areas. Thirdly, the cross-sectional design limited our ability to infer causality. Fourthly, the reliance on spot urine samples collected during health checkups may have introduced measurement errors, as these samples may not have accurately reflected daily urinary excretion levels. Finally, the effect of unmeasured confounding variables, such as socioeconomic status, cannot be ruled out. Our study indicated that parents’ reported salt-related behaviors influenced the estimated salt excretion and Na/K ratio in 4-year-old children.

In conclusion, the frequency of high-sodium food intake was associated with both urinary sodium excretion and the Na/K ratio. However, some unexpected associations were observed between salt-related behaviors and the Na/K ratio. Further research is needed to clarify these associations, particularly in young children, to support their application in clinical practice and public health surveys.

## Abbreviations

BMI: body mass index
CI: confidence interval
IQR: interquartile range
*β*: unstandardized coefficient
Na: sodium
K: potassium

## Supplementary information

**Additional file 1:** **Supplemental Table 1.** Participants’ characteristics. **Supplemental Table 2.** Frequency of responses for each item on the salt check sheet.

## Declarations

### Availability of data and material

Data cannot be shared due to ethical restrictions.
