# Supplementary material for: Association between parent-reported salt-related behaviors and estimated urinary salt excretion: a cross-sectional study of health checkups in 4-year-old children
Source: Environ Health Prev Med. 2025 May 24;30:39. doi: 10.1265/ehpm.25-00076 (PMC12127078; doi:10.1265/ehpm.25-00076)
Supplement: Supplementary file 1 — Additional file 1: Supplemental Table 1. Participants’ characteristics. Supplemental Table 2. Frequency of responses for each item on the salt check sheet. [file ehpm-30-039-s001.docx]

| Supplemental Table 1. Participants' characteristics | | | |  |  |
| --- | --- | --- | --- | --- | --- |
| Variables | Total, n=109 | Boys, n=49 | Girls, n=60 | P value^d^ |  |
| Age month^a^, months | 54 (53, 55) | 54 (53, 55) | 54 (53, 55) | 0.94 |  |
| Year of survey^b^ |  |  |  | 0.71 |  |
| 2022 | 60 (55.0) | 26 (53.1) | 34 (56.7) |  |  |
| 2023 | 49 (45.0) | 23 (46.9) | 26 (43.3) |  |  |
| Height^c^, cm | 102.6 (4.6) | 103.1 (4.6) | 102.2 (4.6) | 0.30 |  |
| Weight^c^, kg | 16.2 (1.8) | 16.2 (1.7) | 16.2 (1.9) | 0.84 |  |
| Body mass index^c^, kg/m^2^ | 15.4 (1.1) | 15.2 (0.9) | 15.5 (1.1) | 0.09 |  |
| Weight status^b^ |  |  |  | 0.52 |  |
| Thinness | 15 (13.8) | 7 (14.3) | 8 (13.3) |  |  |
| Healthy weight | 89 (81.7) | 41 (83.7) | 48 (80.0) |  |  |
| Overweight / obesity | 5 (4.6) | 1 (2.0) | 4 (6.7) |  |  |
| Estimated salt excretion^a^, g/day | 4.4 (3.3, 6.2) | 4.2 (3.2, 5.8) | 4.8 (3.2, 6.6) | 0.38 |  |
| Sodium to potassium ratio^a^ | 2.3 (1.4, 3.3) | 2.2 (1.3, 3.5) | 2.4 (1.4, 3.2) | 0.91 |  |
| ^a^Median (interquartile range); ^b^number (%); ^c^Mean (standard deviation) | | | | |  |
| ^d^T-test was conducted for normally distributed data, Chi-square test, for categorical data, and Mann–Whitney U test, for non-normally distributed data. | | | | |  |
|  |  |  |  |  |  |

| Supplemental Table 2. Frequency of responses for each item on the salt check sheet | | | | | |
| --- | --- | --- | --- | --- | --- |
| Salt check‐sheet items | | Total, n=109 | Boys, n=49 | Girls, n=60 | P value^a^ |
| **Frequency of high‐salt diet intake** | |  |  |  |  |
| *Miso* soup, other soup, etc. | |  |  |  | 0.03 |
|  | More than 2 bowls a day | 14 (12.8) | 6 (12.2) | 8 (13.3) |  |
|  | About 1 bowl a day | 48 (44.0) | 28 (57.1) | 20 (33.3) |  |
|  | Two-three bowls a week | 38 (34.9) | 14 (28.6) | 24 (40.0) |  |
|  | Hardly eat | 9 (8.3) | 1 (2.0) | 8 (13.3) |  |
| Pickles, pickled plums, etc. | |  |  |  | 0.47 |
|  | More than twice a day | 2 (1.8) | 2 (4.1) | 0 (0) |  |
|  | About once a day | 7 (6.4) | 3 (6.1) | 4 (6.7) |  |
|  | Two-three times a week | 29 (26.6) | 13 (26.5) | 16 (26.7) |  |
|  | Hardly eat | 71 (65.1) | 31 (63.3) | 40 (66.7) |  |
| Fish‐paste products such as *chikuwa* and *kamaboko* | |  |  |  | 0.03 |
|  | Eat frequently | 4 (3.7) | 3 (6.1) | 1 (1.7) |  |
|  | Two-three times a week | 37 (33.9) | 22 (44.9) | 15 (25.0) |  |
|  | Hardly eat | 68 (62.4) | 24 (49.0) | 44 (73.3) |  |
| Opened and dried horse mackerel, *mirin*‐seasoned dried fish, salted salmon, etc. | |  |  |  | 0.09 |
|  | Eat frequently | 3 (2.8) | 3 (6.1) | 0 (0) |  |
|  | Two-three times a week | 36 (33.0) | 18 (36.7) | 18 (30.0) |  |
|  | Hardly eat | 70 (64.2) | 28 (57.1) | 42 (70.0) |  |
| Ham or sausage | |  |  |  | 0.16 |
|  | Eat frequently | 17 (15.6) | 11 (22.4) | 6 (10.0) |  |
|  | Two-three times a week | 69 (63.3) | 30 (61.2) | 39 (65.0) |  |
|  | Hardly eat | 23 (21.1) | 8 (16.3) | 15 (25.0) |  |
| Noodles such as *udon* and *ramen* | |  |  |  | 0.82 |
|  | Almost every day | 1 (0.9) | 0 (0) | 1 (1.7) |  |
|  | 2 or 3 bowls a week | 37 (33.9) | 16 (32.7) | 21 (35.0) |  |
|  | Less than once a week | 69 (63.3) | 32 (65.3) | 37 (61.7) |  |
|  | Don't eat | 2 (1.8) | 1 (2.0) | 1 (1.7) |  |
| *Senbei*, *okaki*, potato chips, etc. | |  |  |  | 0.91 |
|  | Eat frequently | 14 (12.8) | 7 (14.3) | 7 (11.7) |  |
|  | Two-three times a week | 49 (45.0) | 22 (44.9) | 27 (45.0) |  |
|  | Hardly eat | 46 (42.2) | 20 (40.8) | 26 (43.3) |  |
| **Additional seasoning, frequency of eating out, and home‐meal replacement** | |  |  |  |  |
| Frequency of seasoning with soy sauce, other sauces, etc. | |  |  |  | 0.73 |
|  | Season frequently (almost each meal) | 3 (2.8) | 2 (4.1) | 1 (1.7) |  |
|  | Once a day | 22 (20.2) | 10 (20.4) | 12 (20.0) |  |
|  | Season sometimes | 62 (56.9) | 29 (59.2) | 33 (55.0) |  |
|  | Don't season | 22 (20.2) | 8 (16.3) | 14 (23.3) |  |
| Consumption of *udon*, *ramen*, or other soups | |  |  |  | 0.14 |
|  | An entire bowl | 4 (3.7) | 0 (0) | 4 (6.7) |  |
|  | About half a bowl | 22 (20.2) | 13 (26.5) | 9 (15.0) |  |
|  | Some | 42 (38.5) | 17 (34.7) | 25 (41.7) |  |
|  | Litte | 41 (37.6) | 19 (38.8) | 22 (36.7) |  |
| Eating out or having convenience‐store‐bought *bento* (lunch plate) for lunch | |  |  |  | 0.66 |
|  | Almost every day | 4 (3.7) | 2 (4.1) | 2 (3.3) |  |
|  | About 3 times a week | 9 (8.3) | 3 (6.1) | 6 (10.0) |  |
|  | About once a week | 49 (45.0) | 20 (40.8) | 29 (48.3) |  |
|  | No | 47 (43.1) | 24 (49.0) | 23 (38.3) |  |
| Eating out or having ready‐made side dishes for dinner | |  |  |  | 0.42 |
|  | Almost every day | 2 (1.8) | 2 (4.1) | 0 (0) |  |
|  | About 3 times a week | 11 (10.1) | 4 (8.2) | 7 (11.7) |  |
|  | About once a week | 55 (50.5) | 24 (49.0) | 31 (51.7) |  |
|  | No | 41 (37.6) | 19 (38.8) | 22 (36.7) |  |
| **Taste of homemade dishes, amount of food** | |  |  |  |  |
| Taste of homemade dishes: comparison with those eaten out | |  |  |  | 0.72 |
|  | Heavily salted | 3 (2.8) | 2 (4.1) | 1 (1.7) |  |
|  | The same | 49 (45.0) | 21 (42.9) | 28 (46.7) |  |
|  | Lightly salted | 57 (52.3) | 26 (53.1) | 31 (51.7) |  |
| Amount of food | |  |  |  | 0.01 |
|  | More than others | 13 (11.9) | 3 (6.1) | 10 (16.7) |  |
|  | The same as others | 86 (78.9) | 45 (91.8) | 41 (68.3) |  |
|  | Less than others | 10 (9.2) | 1 (2.0) | 9 (15.0) |  |
| ^a^Chi-square test was conducted. | | | | | |
